# Deep‐Learning Driven Identification of Novel Antimicrobial Peptides

**DOI:** 10.1002/chem.202501918

**Published:** 2025-08-13

**Authors:** Silvia Arino, Gianmattia Sgueglia, Linda Leone, Rosario Oliva, Pompea Del Vecchio, Gerald Larrouy‐Maumus, Angela Lombardi, Alfonso De Simone, Flavia Nastri

**Affiliations:** ^1^ Department of Chemical Sciences University of Napoli Federico II via Cintia 26 Napoli 80126 Italy; ^2^ Centre for Bacterial Resistance Biology, Department of Life Sciences, Faculty of Natural Sciences Imperial College London London AZ SW7 2 UK; ^3^ Department of Pharmacy University of Napoli Federico II via D. Montesano 49 Napoli 80131 Italy

**Keywords:** antimicrobial peptides, biological assays, biophysical study, computational peptide design, deep learning

## Abstract

In this study, we report the identification of novel antimicrobial peptides (AMPs) via a machine learning‐driven pipeline. Short Trp‐rich peptide sequences were obtained using the HydrAMP deep learning (DL) algorithm, followed by the in silico screening for antimicrobial activity via the AMPlify DL model. Three candidates, namely AMP1, AMP2, and AMP3, were selected for synthesis and experimental validation. The antimicrobial activity was evaluated in vitro against a panel of Gram‐positive and Gram‐negative bacterial strains. Among them, AMP3 demonstrated the broader antibacterial spectrum. To investigate the mechanisms of action, we conducted detailed biophysical analyses of AMP3 interaction with liposomal models of bacterial membranes. The data revealed significant perturbation of membrane bilayer stability, supporting the proposed membrane‐targeting activity of AMP3. Overall, our results underscore the potential of DL approaches for the accelerated discovery and mechanistic characterization of novel AMPs.

## Graphical abstract

Using an AI‐driven pipeline that couples HydrAMP sequence generation with AMPlify activity scoring, we evolved a short tryptophan‐rich parent peptide into a mini‐library of analogues, screened them in silico, and validated the top hits experimentally. The lead candidate, AMP3, shows high antimicrobial activity and membrane‐disruptive ability, demonstrating the power of DL and rational peptide engineering to accelerate antimicrobial peptide discovery.

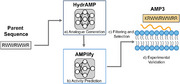

## 1 Introduction

The development of Artificial Intelligence (AI) models has strongly propelled the field of peptide and protein design.^[^ ^1^, ^2^ ^]^ Powerful AI tools are available to decipher complex sequence‐structure‐function relationships thus speeding up the development of novel biomolecules with a large variety of applications including diagnostics, medicine, catalysis, and material science.^[^ ^3^, ^4^, ^5^, ^6^, ^7^, ^8^ ^]^ Deep learning (DL) currently represents a major component of AI. The vast number of DL architectures and algorithms enables the analysis of large, complex datasets comprising peptide sequences, structural descriptors, physicochemical properties, and bioactivity profiles.^[^ ^9^ ^]^ Starting from these large‐scale datasets, DL models can predict the structural and functional properties of de novo proteins and peptides with remarkable accuracy, thus streamlining the design process.^[^ ^10^, ^11^, ^12^ ^]^ Furthermore, the high‐throughput ability of DL algorithms facilitates the exploration of the vast chemical space of peptide sequences, enabling the generation of novel peptides with enhanced functions tailored to specific applications.

One significant application of DL deals with the definition of novel antimicrobial peptides (AMPs).^[^ ^13^, ^14^ ^]^

AMPs, also known as host defense peptides, range in length from 10 to 50 amino acids and exhibit bactericidal, fungicidal, viricidal, and even anticancer properties at low concentrations by acting rapidly.^[^ ^15^ ^]^ They are naturally found within the innate immune system of diverse organisms, including microbes, plants, and animals, and are characterized by chemical and structural heterogeneity.^[^ ^16^ ^]^ Compared to conventional antibiotics, AMPs are less likely to cause bacterial resistance as their antibacterial effects are typically dependent on their initial interaction with cellular membranes, which makes them powerful molecules for combating antimicrobial resistance.^[^ ^17^ ^]^ However, the application of AMPs as therapeutics has been mostly limited by their cytotoxicity and susceptibility to proteases.^[^ ^18^ ^]^ Therefore, there is growing interest in obtaining novel AMP with improved features and fewer drawbacks. Increased stability toward proteases has been obtained through several strategies, including cyclization, acetylation, glycosylation and incorporation of unnatural amino acids into natural or designed peptide sequences.^[^ ^19^, ^20^, ^21^, ^22^, ^23^, ^24^, ^25^, ^26^ ^]^ In addition, the selectivity and potency toward the pathogens can be improved by designing new peptides based on a deep understanding of sequence‐function relationships, investigating their specific mode of action. Indeed, AMPs have been proposed to act through a number of different mechanisms, encompassing membrane alterations, direct inhibition of specific metabolic pathways, or interference with cellular components of the pathogenic organism.^[^ ^27^, ^28^ ^]^

Most AMPs targeting membrane bilayers are typically enriched in cationic amino acids (Arg, Lys, His).^[^ ^29^ ^]^ Further, they share an amphipathic structure, in which cationic and hydrophobic residues, as Phe, Trp, Ile, and Leu, are clustered in different spatial regions.^[^ ^30^ ^]^ Cationic residues are involved in the interaction with negatively charged moieties on the bacterial membranes, such as lipopolysaccharide (LPS) for Gram‐negatives or lipoteichoic acid (LTA) for Gram‐positives.^[^ ^27^, ^31^ ^]^ Further, hydrophobic residues favor AMPs integration within the membrane, initiating various deleterious actions for the pathogen, ultimately leading to the inhibition of bacterial growth or cell death. Specifically, many antimicrobial sequences present significant abundance in Arg and Trp residues, which have shown optimal characteristics for membrane binding and disruption.^[^ ^30^, ^32^ ^]^ A correlation between the number and position of Trp residues into the sequence with the cell‐penetrating and antibacterial properties of natural and de novo AMPs has been reported.^[^ ^33^ ^]^ Besides its hydrophobicity, Trp is characterized by a notable quadrupolar moment, thus forming π‐cation interaction with the positively charged Arg side‐chain. This interaction supports membrane insertion by enabling Trp to pierce the bilayer interface and facilitate Arg penetration into the hydrophobic core.^[^ ^30^ ^]^ In addition to charge and amino acid composition, a crucial aspect determining the effectiveness of AMPs is sequence length, with optimal activity observed in peptides containing around ten residues.^[^ ^34^ ^]^ In a recent study,^[^ ^34^, ^35^ ^]^ all possible combinations of Arg and Trp residues up to seven residues long peptides, were screened to investigate the effects on the antimicrobial activity of sequence length, Trp content, and the relative positions of Arg and Trp. Peptides with a slightly higher Trp content compared to Arg exhibited the best activity. Further, activity increased when the Trp were organized in doublets or triplets. This result was attributed to increased membrane perturbation caused by the proximity of two or three adjacent tryptophan residues.^[^ ^36^ ^]^

In this context, we report the application of two DL algorithms, HydrAMP and AMPlify,^[^ ^37^, ^38^ ^]^ for the development of novel Trp‐rich AMP sequences with in silico predicted antimicrobial activity. Validation of the computational design was performed experimentally on the synthesized sequences, with a first focus on the determination of the peptide antimicrobial activity on different bacterial strains. In addition, a detailed biophysical characterization on lipid vesicles as models of bacterial membranes was carried out on the peptide sequence with the broadest‐spectrum antibacterial activity, to shed light on its mechanism of action.

## 2 Results and Discussion

### 2.1 Peptide Selections and Antimicrobial Activity

**Figure 1:**
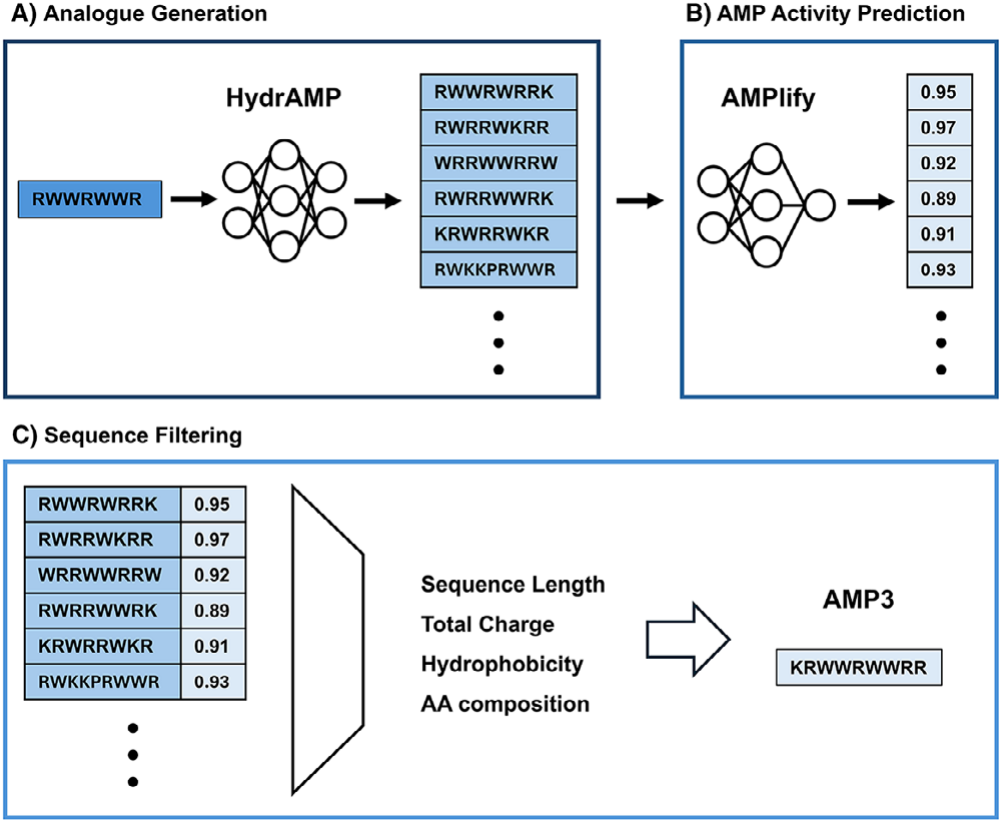
Overview of the AMP design process used in this work. A) HydrAMP generation of new peptide sequence starting from the input sequence. B) Antimicrobial activity prediction by AMPlify. C) Ranking of candidates AMPs on the basis of the predicted activity and empirical filtering, resulting in a refined pool of sequences.

Peptide design and selection started with the identification of minimal peptide sequences housing the essential features for antimicrobial activity. To this end, the peptide series composed exclusively of Arg and Trp residues developed by Dobson et al.^[^ ^34^ ^]^ was deemed suitable to our purposes, as these sequences are short and characterized by a repetitive alternation of hydrophobic and cationic residues, a motif recognized as a key determinant of AMP activity. We used one of the most active sequences reported,^[^ ^34^ ^]^ the Ac‐RWWRWWR‐NH_2_ heptapeptide, as a prototype for generating novel AMPs by the analogue generation protocol of the HydrAMP model (Figure 1).^[^ ^37^ ^]^

Prior to HydrAMP‐based sequence generation, the prototype sequence was rationally modified by introducing a Lys residue at the N‐terminus, retained as a free amine group. This modification yielded the final sequence KRWWRWWR‐NH_2_, hereafter referred to as AMP1. The inclusion of Lys was intended to provide the sequence with functional groups for potential downstream derivatization, such as branched or immobilized AMP‐based materials. Approximately 1000 new sequences were generated from the prototype peptide AMP1, without imposing constraints on improved antimicrobial probability with respect to the original sequence.

These sequences were subsequently evaluated using the AMPlify model,^[^ ^38^, ^39^ ^]^ which predicted all to be in silico putative AMPs. This initial set of sequences was narrowed down through an empirical selection, by applying the following filters. Peptide length was restricted to 8–10 residues, with an overall charge between + 4 and + 10. Sequences bearing sulfur‐containing amino acids (methionine and cysteine) were excluded as they would be susceptible to oxidation. To prevent synthetic difficulties in producing the peptides, sequences containing three or more consecutive tryptophan residues or branched amino acids were also eliminated.^[^ ^40^, ^41^ ^]^ Finally, only sequences possessing an amphiphilic profile were considered, as this behavior is often associated with effective bacterial membrane interaction.^[^ ^42^ ^]^ This selection process afforded a final list of 36 peptide sequences, ranked on the basis of their AMPlify probability scores (see Table S1).

The top‐scoring sequence corresponds to the prototype AMP1 sequence with an extra Arg residue at the C‐terminus, whereas sequence No. 12 corresponds to AMP1 with an extra Gln at the C‐terminus. All sequences show probability scores far above the threshold of 0.5 used by AMPlify to distinguish AMPs from non‐AMPs, and clustered above 0.9. This result was somewhat expected as the lack of experimental data for the antimicrobial activity on very similar sequences strongly affects DL model training, thus limiting AMPlify performances in differentiating them. However, small differences in antimicrobial probability among the sequences can be highlighted by looking at the AMPlify log‐scaled score (Table S1). Interestingly, the two most similar sequences, namely sequence No. 1 and No. 12 differing only for the C‐terminal residue, show AMPlify log‐scaled scores of 56.23 and 43.46, respectively (Table 1). In order to verify whether the predicted log scaled AMPlify scores correspond to different antimicrobial activities, we chose sequence No. 1 and No. 12, named AMP2 and AMP3, respectively, for experimental testing.

**Table 1:** Prototype and HydrAMP generated peptide sequences selected for this study, along with their log scaled scores as predicted by AMPlify.

NAME	SEQUENCE	AMPlify Log Scaled Score
AMP1	**K‐R‐W‐W‐R‐W‐W‐R‐ NH_2_**	58.10
AMP2	**K‐R‐W‐W‐R‐W‐W‐R**‐**Q‐ NH_2_**	43.46
AMP3	**K‐R‐W‐W‐R‐W‐W‐R**‐**R–NH_2_**	56.23

The prototype and the two HydrAMP generated peptide sequences were synthesized by SPPS, purified to homogeneity and their identity was confirmed by ESI‐MS analysis (see supporting Figures S1–S6).

The antimicrobial activity of the three peptides was investigated against both Gram‐negative (*E. coli, P. aeruginosa*) and Gram‐positive (S*. aureus*) bacterial strains, by determining the minimum inhibitory concentration (MIC_50_) leading to no visible bacterial growth (Table 2). Among the three peptides, AMP3 exhibited the most potent activity against both *E. coli* and *P. aeruginosa*, exhibiting MIC_50_ values of 15 µM and 125 µM, respectively. While *Escherichia* coli and *Pseudomonas aeruginosa* are both Gram‐negative bacteria, they vary by cell envelope permeability which can contribute to the tolerance and resistance to some antimicrobials.^[^ ^43^ ^]^ For example, *P. aeruginosa* possesses unique outer membrane proteins and efflux pumps that contribute to its antibiotic resistance. As well, the composition of the endotoxins or LPS from *E. coli* is different from the one found in *P. aeruginosa* contributing to changes in cell envelope permeability.^[^ ^44^, ^45^ ^]^ These features could potentially account for the MIC_50_ values observed toward the two Gram‐negative bacteria.

**Table 2:** MIC_50_ values of the novel peptides against the reported bacterial strains.

Gram‐negative strains	AMP1(µM)	AMP2(µM)	AMP3(µM)
*E. coli*	31	62	15
*P. aeruginosa*	250	250–500	125
**Gram‐positive strains**			
*S. aureus*	31	‐	31

When analyzing the antimicrobial activity against the Gram‐positive *S. aureus*, AMP1 and AMP3 were found comparable, with MIC_50_ values of 31 µM. Finally, the less active sequence against *E. coli* and *P. aeruginosa* was AMP2, (MIC_50_ 62 and 500 µM, respectively) showing no detectable activity toward *S. aureus*.

The antimicrobial activity results provided compelling validation of the DL‐based computation pipeline. Indeed, AMP2 revealed to be the worst AMP sequence, in line with the AMPlify prediction. AMP3 sequence was identified as a leading compound, for its broad‐spectrum antimicrobial properties, being effective with low MIC_50_ values toward both Gram positive and Gram‐negative bacteria. Therefore, it was selected for further biophysical studies on model membranes to gain information on its mechanism of action.

### 2.2 Biophysical Study on Bacterial Model Membranes

**Figure 2:**
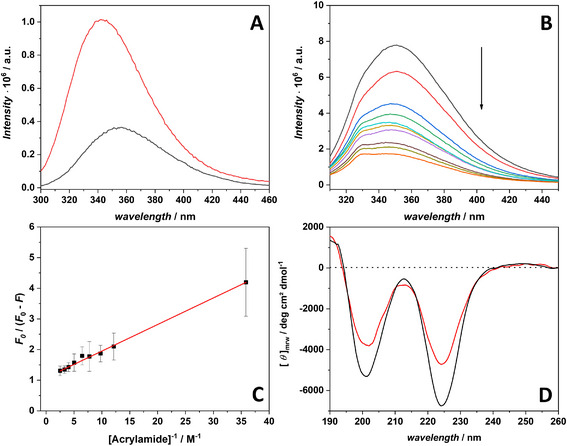
A) Fluorescence emission spectra of AMP3 (2 µM) in the absence (black line) and presence (red line) of POPE/POPG LUVs (550 µM). B) Fluorescence emission spectra of AMP3 (3 µM) mixed with a lipid suspension of POPE/POPG LUVs at a total lipid concentration of 40 µM, when tritated with acrylamide. The arrow indicates the direction of increasing concentration of acrylamide showing the quenching of fluorescence emission. C) Modified Stern‐Volmer plot obtained using the data reported in panel B. The red line is the best fit of experimental data according to the modified Stern‐Volmer equation described in the Supporting Information. D) Far‐UV CD spectra of AMP3 peptide (60 µM) in neat buffer (black line) and in the presence of POPE/POPG LUVs vesicles (red line) at a total lipid concentration of 600 µM. All the experiments were performed in 10 mM phosphate buffer, pH 7.4 at the temperature of 25 °C.

The interaction of AMP3 with 1‐palmitoyl‐2‐oleoyl‐sn‐glycero3‐phosphoethanolamine (POPE) and 1‐palmitoyl‐2‐oleoyl‐sn‐glycero‐3‐phospho‐10 ‐rac‐glycerol (POPG) liposomes (POPE/POPG 7/3 mol/mol) as mimics of bacterial membranes, was investigated via biophysical techniques. The binding ability of AMP3 to the selected bacterial model membrane was first evaluated by Trp fluorescence emission measurements. The emission spectra of the peptide AMP3 in the absence and in presence of 550 µM lipid vesicles, collected in Figure 2A revealed interesting features. Indeed, a blue shift of the emission maximum of about 13 nm coupled with a strong increase in the fluorescence intensity was observed. These findings show that: i) AMP3 can interact with the bacterial model membrane; ii) a change toward a more hydrophobic environment of the tryptophan residues is occurring, suggesting a partial penetration of the peptide into the membrane. To determine the mole fraction partition constant (*K*_x_), a key parameter indicating the strength of peptide–membrane interaction, the established procedure^[^ ^46^ ^]^ has been followed where a peptide solution at fixed concentration (6 µM) was titrated with a suspension of POPE/POPG vesicles, and changes in the fluorescence emission of the Trp residues were evaluated. Unfortunately, this approach proved to be unfeasible, as fluorescence intensity at low lipid‐to‐peptide (L/P < 8) progressively decreased over time, whereas it remained stable at L/P > 10. This signal instability hampers the accurate construction of a binding curve, since experimental data at low L/P ratios were not reproducible. A possible explanation is that, at low lipid concentrations, the peptide induces the liposome aggregation, as previously observed for the interaction of the peptide CA(1–7)M(2–9) with PE/PG vesicles.^[^ ^47^ ^]^ In order to determine the *K*_x_ value, alternative fluorescence quenching experiments were planned using acrylamide as a quencher.

By using this strategy, it is possible to evaluate the fraction of unbound peptide accessible to the quencher (*f*_a_), by analyzing the data with the modified Stern‐Volmer equation,^[^ ^48^ ^]^ which in turn allows the determination of the value of *K*_x_ (see the Supporting Information for details). The reliability of this method was herein ascertained using a reference peptide (Figure S7). The fluorescence emission spectra obtained upon titrating with acrylamide a peptide solution of AMP3 (3 µM) mixed with a suspension of POPE/POPG vesicles (40 µM total lipids concentration) are reported in Figure 2B. Figure 2C reports the modified Stern‐Volmer plot, obtained from the experimental data shown in Figure 2B.This experiment was also repeated with the same AMP3 concentration (3 µM) at a higher lipid concentration (150 µM). The corresponding modified Stern‐Volmer plot is shown in Figure S8. The fraction of unbound peptide, *f*_a_ values, and the calculated *K*_x,_ obtained from the two explored concentrations, are reported in Table S2. The obtained average *K*_x_ value of (2.0 ± 0.4) 10^5^ indicates a strong interaction between AMP3 and the simplified bacterial model membranes, as it is higher than the average value reported for most peptides studied in literature (around 10^4^).^[^ ^49^ ^]^

Far‐UV CD spectroscopy was also employed to analyze the conformation of AMP3 peptide in solution and upon binding with model membranes. The CD spectrum of AMP3 in buffer at pH 7.4 (Figure 2D, black line) is characterized by two minima around 225 nm and 200 nm. The negative band at 225 nm prompted us to assign a turned conformation to the peptide, in comparison with literature data.^[^ ^50^, ^51^ ^]^ This band is likely due to the contribution of Trp transitions^[^ ^52^, ^53^ ^]^ as frequently observed in Trp‐rich peptides.^[^ ^21^, ^54^, ^55^ ^]^ This dichroic signal can be either positive or negative, depending on the backbone conformation around the Trp residues,^[^ ^52^ ^]^ with a negative band around 230 nm assigned to turned structures.^[^ ^50^, ^51^ ^]^ The CD spectrum of AMP3 in the presence of POPE/POPG vesicles at L/P ratio of 10 (Figure 2D, red line) shows no drastic changes in the shape, but only small changes in the intensities of the two bands. This observation confirms that AMP3 can interact with model membranes and suggests small conformational changes imposed by membrane binding.

### 2.3 Effects of AMP3 Peptide on Bilayer Stability

**Figure 3:**
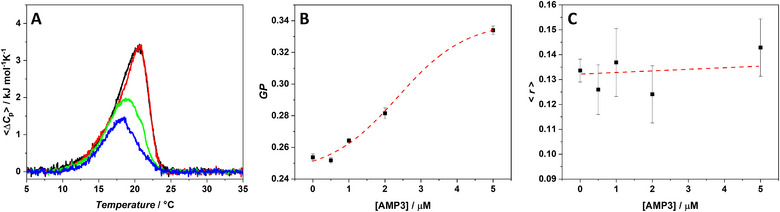
A) DSC thermograms of POPE/POPG (7/3 mol/mol) MLVs at a total lipid concentration of 1 mM in the absence (black line) and in the presence of AMP3 peptide at the lipid‐to‐peptide ratio of: 50 (red line), 25 (green line) and 10 (blue line). B) Generalized polarization (*GP*) of the probe Laurdan embedded in POPE/POPG LUVs as function of AMP3 concentration. C) Fluorescence anisotropy (< *r* >) of DPH in POPE/POPG unilamellar vesicles as function of peptide concentration. The fluorescence experiments were performed at the temperature of 25 °C. The red dashed lines do not represent data fitting but are intended as a guide for the eye.

The effect of AMP3 peptide on POPE/POPG bilayers stability was explored by differential scanning calorimetry (DSC). Figure 3A reports the DSC curve of POPE/POPG multilamellar vesicles (MLVs) in the absence and presence of AMP3 (at lipid‐to‐peptide ratio of 50, 25, and 10). The thermodynamic parameters associated with the gel‐to‐liquid phase transition of the liposomes are reported in Table 3.

**Table 3:** Thermodynamic parameters associated to the gel‐to‐liquid phase transition of POPE/POPG MLVs obtained by means of differential scanning calorimetry.

System	*T*_m_ / °C	^[^ ^a^ ^]^Δ*H*_m_ / kJ mol^−1^
POPE/POPG	20.6 ± 0.1	19.6 ± 2.0
+ AMP3 at L/P = 50	20.9 ± 0.1	18.5 ± 1.9
+ AMP3 at L/P = 25	18.7 ± 0.3	12.3 ± 1.2
+ AMP3 at L/P = 10	18.5 ± 0.3	8.1 ± 0.8

^[a]^ Normalization per total moles of lipids.

The DSC curve of POPE/POPG vesicles is centered at 20.6 °C and it is characterized by an enthalpy change of 19.6 kJ mol^−1^, in good agreement with previously reported data.^[^ ^56^ ^]^

Upon addition of AMP3 at L/P = 50, a slight increase of the main transition temperature (*T*_m_) was observed, while the enthalpy change (Δ*H*_m_) remained roughly constant within the experimental error. In addition, the peak became sharper, suggesting an increase of transition cooperativity. These effects can be attributed to the AMP3 preferential interaction with the negatively charged headgroups of POPG lipids (thus on the surface), which in turn will promote the formation of a “purer” PE‐rich domain that transits at higher *T*_m_ and more cooperatively. By increasing the peptide concentration at L/P = 25, a significant decrease of both the *T*_m_ and Δ*H*_m_ was detected. Remarkably, the DSC curve appeared composed of at least two partially overlaid transitions, indicating that lipid domains are formed within the bilayer, as observed for several cationic peptides.^[^ ^57^, ^58^, ^59^ ^]^ The peak centered at lower *T*_m_ could be due to a POPG‐rich domain. Instead, the high‐temperature peak could be attributed to a POPE‐rich domain (please, note that the *T*_m_ of POPE and POPG are ∼24 °C and ∼‐5 °C, respectively). Of note, the enthalpy change is lower with respect to that detected for pure liposomes and at L/P = 50 suggesting the insertion (at some extent) of the peptide into the membrane that disrupts the lipid packing, as also observed for poly‐Lys peptide with PG membranes.^[^ ^60^ ^]^ At the highest peptide concentration explored (L/P = 10), a further reduction of the Δ*H*_m_ was observed, whereas no change in the value of *T*_m_ occurred. Surprisingly, the higher‐temperature peak previously observed disappeared. This can be due to the accumulation and insertion of peptide molecules into the PE‐rich domain that disrupts the lipid packing. Even though it could be surprising that the cationic AMP3 affects more prominently the PE‐rich domain than the PG‐rich one, it is important to outline that i) both lipids are present in the two domains, thus the peptide can establish interactions everywhere on the membrane and ii) the PE lipids are characterized by a smaller headgroup, which promotes a negative curvature to the membrane facilitating the access of the peptide in the bilayer. From the DSC data reported above, an insertion of AMP3 within the bilayer can be inferred, according to the fluorescence results. However, it is not clear if the peptide can penetrate deeply into the hydrophobic core of the bilayer or it remains confined close to the lipid headgroups region.

To shed light on the AMP3‐membrane interaction, complementary fluorescence experiments using Laurdan and DPH probes, embedded in POPE/POPG vesicles, were performed. In Figures 3B, 3C, the *GP* of Laurdan and DPH anisotropy (< *r* >) as function of peptide concentration are reported, respectively. Laurdan localizes itself with the fluorescent moiety close to the headgroup region of the bilayer, serving as a reporter of this portion of the membrane.^[^ ^61^, ^62^ ^]^ The *GP* value quantifies the degree of order in the membrane and it can vary from 1 to ‐1 when passing from the gel phase (more compact, high degree of order, less hydrated) to the fluid‐like phase (less compact, low degree of order, more hydrated).^[^ ^62^ ^]^ A close inspection of Figure 3B reveals that the *GP* value significantly increases with peptide concentration, passing from 0.25 (no peptide) to 0.33 at 5 µM [AMP3 concentration], which corresponds to L/P = 10. This is clear evidence of the peptide binding and localization at the level of headgroups of lipids. It is possible to hypothesize that, through its interaction, the peptide promotes the dehydration of the surface and then, by partially penetrating into the bilayer, it induces a higher degree of order in this region of the membrane. The DPH probe is highly hydrophobic, thus it partitions deeply in the hydrophobic core of the bilayer. Interestingly, in the explored AMP3 concentration range (up to 5 µM, L/P = 10), no significant changes in DPH anisotropy were observed (Figure 3C), indicating that the interior of the membrane is not perturbed by the peptide. Collectively, all the reported biophysical data suggest that AMP3 affects the microscopic and mesoscopic properties of the bilayer in a concentration‐dependent mode. At low concentration, the peptide is localized on the surface of the bilayer. Increasing its concentration, domains formation takes place: the segregation can facilitate the partial insertion of the peptide into the bilayer, remaining mostly localized at the level of its headgroup regions.

## 3 Conclusion

This study highlights the potential of integrating DL methodologies with experimental validation to accelerate the discovery of novel AMPs. By applying this pipeline, we successfully generated three Trp‐rich peptide sequences that exhibit antimicrobial activity against different bacterial strains. Among the three sequences, AMP3 emerged as the most potent candidate, demonstrating broad‐spectrum efficacy at low micromolar concentrations against both Gram‐positive and Gram‐negative bacteria. Biophysical analyses indeed showed the ability of AMP3 to efficiently integrate into the lipid bilayers, as highlighted by the very high value calculated for the mole fraction partition constant (*K*_x_). This high membrane affinity supports a mode of action involving peptide insertion at the membrane interface, leading to the formation of lipid domains. It is expected that such alterations in membrane properties could severely affect its functionality and influence the properties of other components like proteins, present within the bilayer. ^[^ ^63^, ^64^ ^]^ Our biophysical data suggest that the bacteria membrane may be the main target of the AMP3 peptide. However, it remains possible that AMPs are capable of interacting also with other membrane components (e.g., LTA, LPS), or they can have intracellular targets.^[^ ^21^, ^65^, ^66^ ^]^ Further studies are needed to assess the potential presence of other targets.

Beyond the identification of a promising AMP candidate, this work also contributes valuable structure–activity relationship data for the advancement of DL‐based predictive models, as for example AMPlify. The data we obtained on two very similar short AMP sequences, differing only in the C‐terminal residues that revealed to be crucial for the activity, may indeed increase the effectiveness of DL‐based predictive tools in discriminating among similar sequences.

## 4 Experimental Section

### Computational AMP design and selection

Two DL‐based models, HydrAMP and AMPlify^[^ ^38^ ^]^, were used in combination to generate and select novel AMPs. Starting from the AMP1 prototype peptide (KRWWRWWR‐NH2), candidate sequences were generated using HydrAMP. Initial candidate pool was created using the analogue generation protocol, with 20000 total decoding attempts and *T*_m_ equal to 5.0. A total of ≅ 1000 potential AMPs were obtained from the prototype sequence. Following an empirical selection (see results and discussion) the generated sequences were scored and ranked using AMPlify. The final pool of AMP candidates is reported in (Table S1).

### Peptide synthesis and analysis

All the peptides were synthesized by automatic solid‐phase synthesis using standard Fmoc protocols (see Supporting Information for details). For all the sequences, the N‐terminus was kept free, whereas the C‐terminus was amidated. The peptides were purified to homogeneity via RP‐HPLC, and their identity was ascertained by high‐resolution ESI‐MS (Figures S1–S6). Concentration of peptides in the stock solution was determined by measuring the absorbance at 280 nm, using ε_280 nm_ = 22 400 M^−1^ cm^−1^.^[^ ^67^ ^]^

### Antimicrobial assays

The antimicrobial activity of the peptides was tested against *Escherichia coli* (MC1000), *Pseudomonas aeruginosa* (PA14) and *Staphylococcus aureus* (USA300), laboratory collection. MIC_50_ assays were performed by the broth microdilution method as described elsewhere^[^ ^68^ ^]^ using Mueller Hinton‐II medium (from Sigma Aldrich) as the bacterial growth medium. In brief, a 96‐well microtitre plate was used to prepare a range of AMP concentration in 100 µl of medium via a series of twofold serial dilutions. Plates were then incubated statically at 37 °C for 18 hours, at which point the MIC_50_ was defined as the lowest antibiotic concentration at which there was no visible growth of bacteria.

Experiments were performed in biological and technical triplicates.

### Circular dichroism spectroscopy

Circular dichroism (CD) spectra of peptide AMP3 were recorded at 25 °C on a JASCO J‐815 dicrograph equipped with a thermoregulated cell holder, using a 0.1 cm path‐length quartz cuvette. Spectra were averaged over three scans, from 260 to 190 nm, with a scan speed of 20 nm/minutes, a response time of 4 seconds, and a bandwidth of 2 nm. Peptide samples were prepared in a 10 mM phosphate buffer at pH 7.4, with a peptide concentration of 60 µM. CD spectra were collected both in the absence and presence of LUVs at lipid concentrations of 600uM (see Supporting Information for details on liposome preparation). For each sample, spectra were background subtracted.

### Differential scanning calorimetry

DSC measurements were performed using a nano‐DSC from TA instruments (New Castle, DE, USA). MLVs were used for all DSC experiments since they provide the better resolution of the peak.^[^ ^69^, ^70^ ^]^ The calorimetry vessel was filled with 700 µL of 1 mM vesicle suspension (POPE/POPG 70/30), in the absence or in the presence of the peptide. The reference cell was filled with sodium phosphate buffer. The experiments were performed over a *T*_m_ range of 20–50 °C, at scan rate of 1 °C/minutes. The obtained data were analyzed by means of NanoAnalyze software supplied with the instrumentation. The reported enthalpy changes of lipid phase transitions were normalized by total moles of lipids. Origin software (OriginLab, Northampton, MA, USA) was used to elaborate the data.

### Steady‐state fluorescence spectroscopy

All the fluorescence experiments were performed by means of a Fluoromax‐4 from Horiba Scientific (Edison, USA) at the *T*_m_ of 25 °C using a quartz cuvette with a path length of 1 cm.

## Supporting Information

Supporting Information is available from the Wiley Online Library and includes supplementary methods on peptide synthesis and purification, and biophysical analysis. The authors have cited additional references within the Supporting Information.^[^ ^71^, ^72^, ^73^, ^74^, ^75^ ^]^

## Conflict of Interest

The authors declare no conflict of interest.

## Supporting information



Supplementary Information

## Data Availability

The data that support the findings of this study are available in the supplementary material of this article.
