# Supplementary material for: Deep‐Learning Driven Identification of Novel Antimicrobial Peptides
Source: Chemistry. 2025 Aug 13;31(52):e01918. doi: 10.1002/chem.202501918 (PMC12444733; doi:10.1002/chem.202501918)
Supplement: Supplementary file 1 — Supplementary Information [file CHEM-31-e01918-s001.docx]

Supporting Information

Deep-learning Driven Identification of Novel Antimicrobial Peptides

Silvia Arino,^[a]^ Gianmattia Sgueglia,^[a]^ Linda Leone,^[a]^ Rosario Oliva,^[a]^ Pompea Del Vecchio,^[a]^ Gerald Larrouy-Maumus,^[b]^ Angela Lombardi,^[a]^ Alfonso De Simone,*^[c]^ and Flavia Nastri*^[a]^

[a] Dr. S. Arino, Dr. G. Sgueglia, Dr. L. Leone, Dr. R. Oliva, Prof. P. Del Vecchio, Prof. A. Lombardi, Prof. F. Nastri Department of Chemical Sciences
University of Napoli Federico II
via Cintia 26, 80126, Napoli, Italy
e-mail: flavia.nastri@unina.it

[b] Prof. G. Larrouy-Maumus
Centre for Bacterial Resistance Biology, Department of Life Sciences, Faculty of Natural Sciences,

Imperial College London
London, SW7 2AZ, UK

[c] Prof. A. De Simone

Department of Pharmacy

University of Naples Federico II

via D. Montesano 49, 80131-Naples, Italy

e-mail: alfonso.desimone@unina.it

**Table of contents**

**Supplementary Methods**

- Peptide synthesis and purification
- Biophysical methods
- Determination of the mole fraction partition constant through fluorescence quenching methodology by acrylamide

**Supplementary Tables**

- Table S1: Sequences and antimicrobial scores of the generated peptides.
- Table S2: Fraction of free peptide (*f_a_*) and mole fraction partition constants (*K*_x_) obtained from the analysis of fluorescence quenching data using the modified Stern-Volmer for the AMP3-POPE/POPG systems

**Supplementary Figures**

- Figure S1: HPLC chromatogram of the peptide AMP1
- Figure S2: ESI MS spectrum of pure peptide AMP1
- Figure S3: HPLC chromatogram of the peptide AMP2
- Figure S4: ESI MS spectrum of pure peptide AMP2
- Figure S5: HPLC chromatogram of the peptide AMP3
- Figure S6: ESI MS spectrum of pure peptide AMP3
- Figure S7: (A) Fluorescence emission spectra of LL-III (6 µM) mixed with POPC/POPG (8/2 mol/mol) LUVs at total lipid concentration of 100 µM. The arrow indicates the direction of increasing concentration of acrylamide. (B) The modified Stern-Volmer plot obtained using the fluorescence intensity at 331 nm.
- Figure S8: The modified Stern-Volmer plot obtained from the titration with acrylamide of a solution containing of AMP3 (3 µM) mixed with POPE/POPG vesicles at total lipid concentration of 150 µM.

**Supplementary methods**

**Peptide synthesis and purification**

**Reagents**

Nα-Fmoc-protected amino acids, including Fmoc-Lys, Fmoc-Arg, Fmoc-Trp (Boc), Fmoc-Gln, Fmoc-Ahx, were supplied from Novabiochem (Merck). Coupling reagents such as HATU (1-[Bis(dimethylamino)methylene]-1H-1,2,3-triazolo[4,5-b]pyridinium-3-oxide hexafluorophosphate) and HOBt (1-hydroxybenzotriazole), along with Rink amide resin (0.54 mmol/g of loading substitution), were commercially obtained from Merck. All solvents and reagents DMF (N, N-dimethylformamide) DCM (dichloromethane), N-Methyl-2-pyrrolidone (NMP), N, N-diisopropylethylamine (DIEA), piperidine, trifluoroacetic acid (TFA), and acetonitrile (ACN) used for synthesis and purification were purchased from Romil. Additionally, solvents and reagents for peptide synthesis were reagent grade. Solvents used in HPLC purifications were analytical grade (Super-purity-solvent, SpS – Romil), while solvents with a higher degree of purity (Ultra-purity-solvent, UpS, Romil) were used in mass spectrometry (MS) analyses. All solvents and reagents were used without further purification.

**Solid Phase Peptides Synthesis**

Peptides AMP1, AMP2 and AMP3 were obtained using an ABI 433A peptide synthesizer (Applied Biosystems, Foster City, CA, USA) through standard Fmoc chemistry on a 0.25 mmol scale. The solid support employed was Rink amide resin with a substitution level of 0.54 mmol/g. Amino acids were activated in situ using HATU as the coupling reagent. The cleavage of the peptide from the resin and sidechain deprotection was achieved using a mixture of 95% TFA, 2.5%TIS, 2.5% H_2_O. The reaction was carried out for 1h at 0°C (using an ice bath) followed by 1h at room temperature, under stirring. The resin was then filtered and washed with neat TFA. The excess of TFA was removed under reduced pressure. Subsequently, crude peptides were precipitated in cold diethyl ether and dried under reduced pressure.

**Peptides purification**

The initial purification of the crude peptides was performed by reverse phase flash chromatography, using a Biotage Isolera system. C18 columns were employed, eluted with a linear gradient of H_2_O 0.1% TFA (eluent A) and acetonitrile 0.1% TFA (eluent B) from 5% to 70% of solvent B over 10 column volumes. Subsequent peptide purification was carried out with a Shimadzu LC-8A preparative HPLC system (Shimadzu, Kyoto, Japan) featuring an SPD-M10AV UV-Vis detector. A Reverse Phase (RP) Vydac C18 column (250 cm x 22 mm; 10 µm) was employed, eluting with a linear gradient of H_2_O 0.1% TFA (eluent A) and acetonitrile 0.1% TFA (eluent B) at a flow rate of 23 mL/min. A gradient from 5% to 70% of solvent B was applied for optimal separation from undesired by-products. Peptide purity and identity were evaluated through RP-HPLC-MS analyses using a Shimadzu LC-10ADvp equipped with an SPDM10Avp diode-array detector. Mass spectra were recorded on a Shimadzu LC-MS-2010EV system with ESI interface and a quadrupole mass analyzer. A Vydac C18 column (150 mm x 4.6 mm, 5 µm) was used in all the analyses, by properly scaling the gradients employed for purification. A flow rate of 1 mL/min was used in LC analyses and of 0.5 mL/min in LC-MS analyses. ESI-MS analyses were performed in the positive ion scanning mode, covering the 400–1800 range of m/z.

Peptide samples for biological assays were subjected to counter ion exchange to remove the trifluoroacetate anion. Thus, anionic exchange with chloride was performed by dissolving pure peptides (1 mg/mL) in 10 mM HCl and lyophilized after a 5-minute incubation. This procedure was repeated three times to ensure complete removal of TFA.

**Biophysical methods**

**Vesicles preparation**

Vesicles for various experiments were prepared through a multi-step process. Lipids stock solutions were prepared by weighting appropriate amounts of lipids and dissolving them in a chloroform/methanol (2/1 vol/vol) mixture. These stock solutions were used to prepare liposomes of POPE/POPG (7/3 mol/mol) by mixing appropriate amounts of the two lipids dissolved in the organic mixture. To form lipid films, the organic solvent was removed using a stream of dry nitrogen gas. The lipid films were kept under vacuum for approximately 5 hours to ensure the complete removal of any residual organic solvent. Then, the lipid films were hydrated with a given volume of buffer solution and vigorously vortexed to produce a suspension of Multi Lamellar Vesicles (MLVs). Typically, liposomes suspensions with a total lipid concentration of 5 mM (final volume 1 mL) were prepared. For the preparation of large unilamellar vesicles (LUVs), the MLVs suspensions were extruded, 31 times, through a 100 nm polycarbonate membrane using a mini-extractor (from Avanti Polar Lipid Inc., Alabaster, USA). For the preparation of the labeled vesicles (with the probes Laurdan or DPH), the fluorescence probes dissolved in DMF (Laurdan) or chloroform (DPH) were added to the organic mixture containing lipids and then, the same procedure as described above was followed. For DPH, the lipid-to-probe ratio was 150. Instead, for Laurdan, the lipid-to probe ratio was 30. The resulting LUVs suspensions were subsequently used to study peptide binding.

**Binding Experiments**

In order to evaluate the ability of the peptide AMP3 to interact with POPE/POPG LUVs, two approaches were used. In the first approach, the changes in the fluorescence intensity of the peptide (due to Trp residues) was evaluated by recording emission spectra of the peptides in solutions where the AMP3 concentration was fixed at 6 μM and varying the lipids concentration in the range 0 to ∼1 mM. The excitation wavelength was set to 280 nm, and the emission spectra were collected from 300 to 450 nm. The slit widths for excitation and emission wavelengths were set to 8 nm and 10 nm, respectively. To avoid distortions in the emission spectra due to light scattering induced by liposomes, the titrations were performed using polarizers.^[71, 72]^ However, we found that the data were not reproducible, most likely due to the AMP3-induced liposomes aggregation at low lipid-to-peptide ratio. For this reason, a second approach was followed, and the *K*_x_ was evaluated by performing quenching experiments by using acrylamide (for details, see the paragraph below). The experiments were conducted as follows: a solution of 3 μM of AMP3 was mixed with a vesicles suspension of POPE/POPG LUVs at total lipid concentration of 40 μM and 150 μM. The samples were then titrated with an aqueous solution of acrylamide at 25 wt% where the same amount of peptide and lipids were also present to avoid their dilution during the addition. The emission spectra were recorded from 310 nm to 450 nm, with the excitation wavelength set at 295 nm and using a 1-cm path length quartz cuvette with a magnetic stirrer bar. The fluorescence intensity of the sample in the presence of acrylamide was corrected for its absorbance at the wavelength of excitation, as previously described.^[73]^ All the experiments were performed in 10 mM sodium phosphate buffer, pH 7.4 and at the temperature of 25 °C.

**Laurdan generalized polarization (GP)**

Fluorescence emission spectra of the probe Laurdan embedded in POPE/POPG LUVs were recorded upon excitation at 340 nm and collecting the intensity in the range 390-620 nm. The lipid concentration was 50 μM and the lipid-to-probe ratio was 30. Emission spectra in the presence of AMP3 peptide at concentrations of 0, 0.5, 1, 2, 5 and 10 μM were recorded. The generalized polarization (GP) parameter was evaluated through the equation:

$$GP= \frac{F_{437}-F_{500}}{F_{437}+F_{500}}$$

where *F*_437_ and *F*_500_ were the fluorescence intensities in the Laurdan emission spectra at 437 nm and 500 nm, respectively. All the experiments were performed in 10 mM sodium phosphate buffer, pH 7.4 and at the temperature of 25 °C.

**DPH fluorescence anisotropy**

Fluorescence anisotropy experiments were carried out for the probe DPH embedded in POPE/POPG LUVs. Anisotropy of DPH was evaluated at 427 nm upon excitation at 355 nm. The total lipid concentration was 50 μM and the lipid-to-probe ratio was 150. Experiments were carried out for liposomes in the absence of AMP3 peptide and in its presence at the concentrations of 0, 0.5, 1, 2, 5 and 10 μM. All the experiments were performed in 10 mM sodium phosphate buffer, pH 7.4 and at the temperature of 25 °C.

**Determination of the mole fraction partition constant through fluorescence quenching methodology by acrylamide**

The evaluation of the mole fraction partition constant (*K*_x_) was carried out by performing fluorescence quenching experiments using acrylamide as a quencher. Acrylamide is a water-soluble organic molecule capable to quench the fluorescence emission of Trp residues of a given peptide. More importantly, it cannot partition inside the membrane. Thus, in a given sample containing both peptides and lipids, acrylamide almost exclusively quenches the emission from the free peptide (not bound to the membrane). The peptide can exist in only two forms (bound to the membrane and unbound). The fraction of free peptide and its concentration can be evaluated from the analysis of the data by using a modified version of the Stern-Volmer equation^[74]^

$$\frac{F_{0}}{\Delta F}=\frac{1}{f_{a}K_{a}[Q]}+\frac{1}{f_{a}} (1)$$

In this equation, *F*_0_ is the intensity of the sample in the absence of acrylamide. Instead, ∆*F* = *F* - *F*_0_ is the difference in the intensity of the sample in the presence and absence of acrylamide, respectively. The parameters *K*_a_ and *f*_a_ are the dynamic quenching constant (also denoted as the Stern-Volmer constant) and the fraction of free peptide (accessible to the quencher), respectively. The dynamic quenching constant describes quantitatively the ability of acrylamide to turn off the fluorescence emission of the accessible fluorophore. Instead, [Q] is the concentration of the quencher acrylamide. Thus, from such data analysis, it is possible to evaluate the fraction of free peptide that, multiplied by the total concentration of the peptide gives the concentration of free peptide. Finally, it is possible to calculate the mole fraction partition constant, *K*_x_ the definition:

$$K_{x}=\frac{{[P_{b}]}/{[L]}}{{[P_{f}]}/{[W]}} (2)$$

where [*P_b_*], [*P_f_*], [*L*] and [*W*] are the molar concentrations of the membrane-bound peptide, the free peptide in the aqueous phase, the lipids and water, respectively. Before applying this method to the peptide AMP3, we validated the method on already known and characterized system. In particular, in our previous work ^[75]^ we have estimated the *K*_x_ for the interaction of the peptide LL-III with POPC/POPG (8/2 mol/mol) vesicles. Performing the titration in a conventional way, (i.e. titrating a solution of peptide at fix concentration and varying the lipid concentration) and using polarizers to avoid spectral distortion due to the vesicles-induced light scattering, a *K*_x_ of (4.5 ± 1.0) 10^5^ was determined. To validate our methodology, we tried to estimate *K*_x_ through the quenching experiment. Briefly, a solution of LL-III peptide at 6 µM was mixed with POPC/POPG vesicles at the total lipid concentration of 100 µM was prepared in 10 mM phosphate buffer, pH 7.4 at the total volume of 1 mL. Then, this sample was titrated with a solution of acrylamide (at 25 wt%) and its concentration was varied from 0 to 500 mM. To avoid dilution of the peptide and lipid vesicles, the acrylamide solution contained the same amount of LL-III and lipids. The excitation was set at 295 nm and the emission was collected in the range 310-450 nm. The emission was also corrected for the (small) absorption of acrylamide at this wavelength. In the Figure S7, panel A the recorded fluorescence emission spectra of LL-III mixed with POPC/POPG vesicles are reported. Instead, in the panel B of the Figure S7, a plot of *F*_0_ ⁄ ∆*F* vs. 1 ⁄ [Q] is shown. The fluorescence intensity was evaluated at 331 nm which represents the emission at the maximum in the spectra (λ_max_).

After data analysis, the value of fraction of free peptide accessible to the quencher (*fa*) is 0.612 ± 0.022. Thus, the value of *K*_x_ was determined to be (3.6 ± 0.4) 10^5^, in excellent agreement with the previously reported value. The experiment reported above completely validates our methodology.

**Supplementary Tables**

**Table S1**. The table shows the 36 sequences obtained through machine learning tools that were found to satisfy all required properties (length, composition, charge, hydrophobicity, etc.). The sequences 1^st^ and 12^th^ are the chosen once, respectively named AMP3 and AMP2.

| N° | Sequence | Length | Charge | Probability score | Log-scaled score | Prediction |
| --- | --- | --- | --- | --- | --- | --- |
| 1 | **KRWWRWWRR** | **9** | **5** | **0.999998** | **56.23** | **AMP** |
| 2 | KWWKRWWRR | 9 | 5 | 0.999997 | **54.61** | AMP |
| 3 | RRWWRWWR | 8 | 4 | 0.999991 | **50.49** | AMP |
| 4 | KWWRRWWRR | 9 | 5 | 0.999990 | **50.21** | AMP |
| 5 | KRWWRKWRR | 9 | 6 | 0.999986 | **48.63** | AMP |
| 6 | KRWKRWWRR | 9 | 6 | 0.999977 | **46.40** | AMP |
| 7 | KRRWRWWRR | 9 | 6 | 0.999975 | **45.97** | AMP |
| 8 | KRWWRRWRK | 9 | 6 | 0.999973 | **45.73** | AMP |
| 9 | KRWKRWWRY | 9 | 5 | 0.999972 | **45.53** | AMP |
| 10 | KRWRRWWRR | 9 | 6 | 0.999969 | **45.09** | AMP |
| 11 | KRWWRKWRW | 9 | 5 | 0.999966 | **44.72** | AMP |
| 12 | **KRWWRWWRQ** | **9** | **4** | **0.999955** | **43.46** | **AMP** |
| 13 | KRWWRKWRK | 9 | 6 | 0.999951 | **43.11** | AMP |
| 14 | KRWWRRWRW | 9 | 5 | 0.999950 | **42.98** | AMP |
| 15 | KRWWRRWRQ | 9 | 5 | 0.999933 | **41.77** | AMP |
| 16 | KRWRRWWRY | 9 | 5 | 0.999928 | **41.45** | AMP |
| 17 | KRWWYRWRK | 9 | 5 | 0.999920 | **40.95** | AMP |
| 18 | KRWWRRWK | 8 | 5 | 0.999907 | **40.31** | AMP |
| 19 | KRWWRWRRKW | 10 | 6 | 0.999901 | **40.05** | AMP |
| 20 | KRWWQWWRR | 9 | 4 | 0.999873 | **38.98** | AMP |
| 21 | KRWKRWWRW | 9 | 5 | 0.999866 | **38.74** | AMP |
| 22 | KRWWRWQRR | 9 | 5 | 0.999835 | **37.84** | AMP |
| 23 | KRRWRWWRRR | 10 | 7 | 0.999824 | **37.54** | AMP |
| 24 | KRWRRWWRW | 9 | 5 | 0.999774 | **36.46** | AMP |
| 25 | KRRWRWWRKK | 10 | 7 | 0.999682 | **34.98** | AMP |
| 26 | KRWQRWWRR | 9 | 5 | 0.999602 | **34.00** | AMP |
| 27 | KRRWRWWRKR | 10 | 7 | 0.999576 | **33.72** | AMP |
| 28 | KRRWRWWRHK | 10 | 6 | 0.999568 | **33.65** | AMP |
| 29 | KRWKRWWRKW | 10 | 6 | 0.999549 | **33.45** | AMP |
| 30 | KRWKRWWRQ | 9 | 5 | 0.999538 | **33.35** | AMP |
| 31 | KRWRRWWRKW | 10 | 6 | 0.999406 | **32.26** | AMP |
| 32 | KRRWRWWQR | 9 | 5 | 0.999372 | **32.02** | AMP |
| 33 | KRWRRWWRQ | 9 | 5 | 0.999146 | **30.68** | AMP |
| 34 | KRRWRRWRKR | 10 | 8 | 0.998908 | **29.62** | AMP |
| 35 | KRRWRWWQRR | 10 | 6 | 0.984675 | **18.15** | AMP |
| 36 | KRRWRWWQKR | 10 | 6 | 0.976649 | **16.32** | AMP |

**Table S2.** Fraction of free peptide (*f_a_*) obtained from the analysis of fluorescence quenching data using the modified Stern-Volmer equation for the two explored concentrations. From these values, the mole fraction partition constant (*K*_x_) was calculated. The adjusted R-squared values obtained after data analysis are also reported.

| **System** | ***f_a_*** | ***K*_x_** | ***R*^2^** |
| --- | --- | --- | --- |
| AMP3 + POPE/POPG 40 µM | 0.9244 ± 0.026 | (1.53 ± 0.56) · 10^5^ | 0.9681 |
| AMP3 + POPE/POPG 150 µM | 0.6154 ± 0.008 | (2.37± 0.04) · 10^5^ | 0.9821 |

**Supplementary Figures**

**Figure S1** RP-HPLC chromatogram at λ = 210 nm of pure AMP1 peptide. The desired product is eluted at R_T_ = 13.10 min (purity >98%).

**Figure S2**. ESI MS spectrum of the pure AMP1 peptide. The peaks at m/z 680.05 and 453.90 correspond to the [M+2H^+^]^2+^ and [M+3H^+^]^3+^ ions, respectively. The peak at m/z 737.10 corresponds to the [M+2H^+^+TFA]^2+^ adduct ion. The experimental mass value of 1358.1 Da is consistent with the theoretical mass 1358.62 Da.

**Figure S3** RP-HPLC chromatogram at λ = 210 nm of the pure AMP2 peptide. The desired product is eluted at R_T_ = 13.02 min (purity > 97%).

**Figure S4**. ESI MS spectrum of the pure AMP2 peptide. The peaks at m/z 744.10 and 496.55 correspond to the [M+2H^+^]^2+^ and [M+3H^+^]^3+^ ions, respectively. The peak at m/z 534.55 and 801.25 corresponds to the [M+3H^+^+TFA]^3+^ and [M+2H^+^+TFA]^2+^ adduct ions, respectively. The experimental mass value 1486.5 Da is consistent with the theoretical mass 1485.8 Da.

**Figure S5.** RP-HPLC chromatogram at λ = 210 nm of pure AMP3 peptide. The desired product is eluted at R_T_ = 12.88 min (yield > 98%).

**Figure S6**. ESI MS spectrum of the pure AMP3 peptide. The peak at m/z 505.95 corresponds to the [M+3H^+^]^3+^ ion. The peaks at m/z 408.20, 543.95 and 815.15 represent TFA adduct ions, namely [M+4H^+^+TFA]^4+^, [M+3H^+^+TFA]^3+^ and [M+2H^+^+TFA]^2+^ respectively. The peak at m/z 872.50 corresponds to the [M+2H^+^+2TFA]^2+^ adduct. The experimental mass value 1514.8 Da is consistent with the theoretical mass of 1513.84 Da.


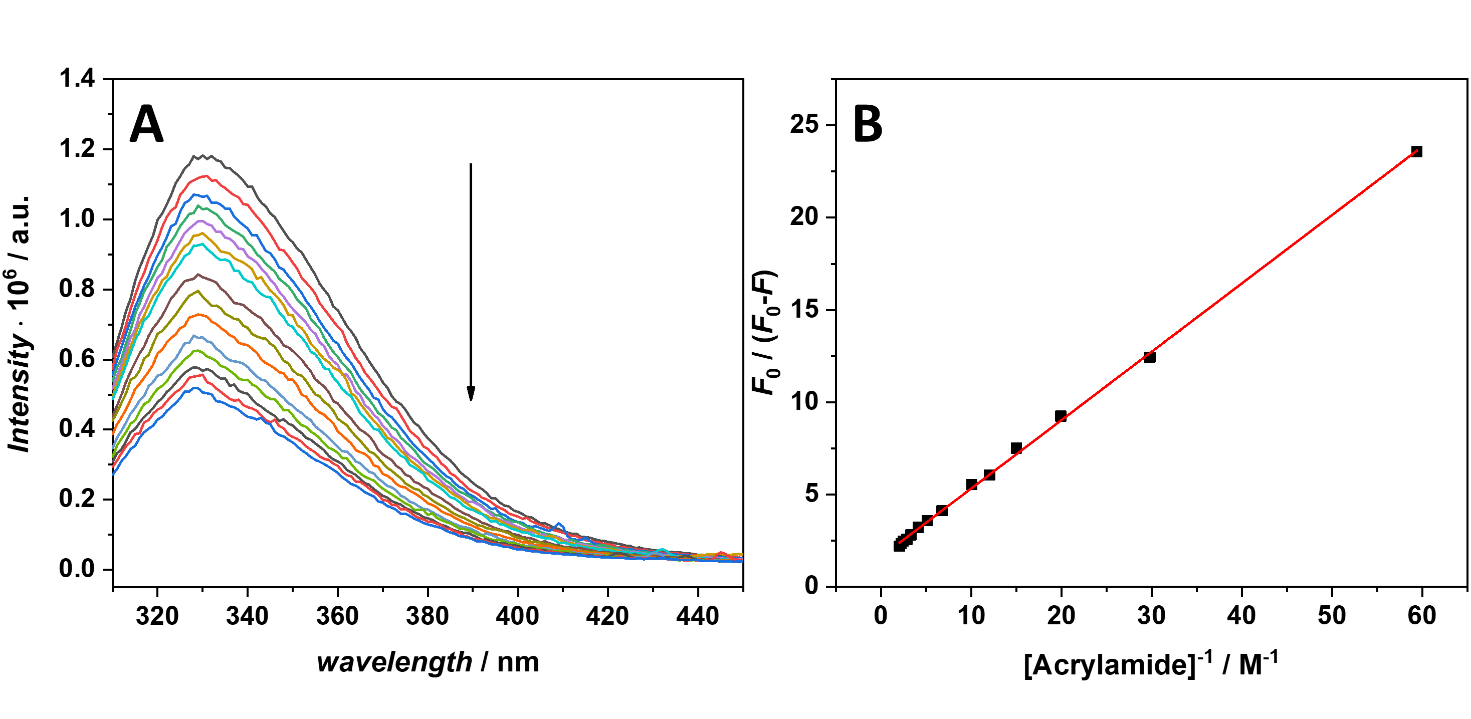


**Figure S7** (A) Fluorescence emission spectra of LL-III (6 µM) mixed with POPC/POPG (8/2 mol/mol) LUVs at total lipid concentration of 100 µM. The arrow indicates the direction of increasing concentration of acrylamide. (B) The modified Stern-Volmer plot obtained using the fluorescence intensity at 331 nm. The red line represents the best fit of experimental data according to equation 1. The experiment was performed at the temperature of 25 °C in 10 mM phosphate buffer, pH 7.4 and, using a quartz cuvette with a path length of 1 cm.


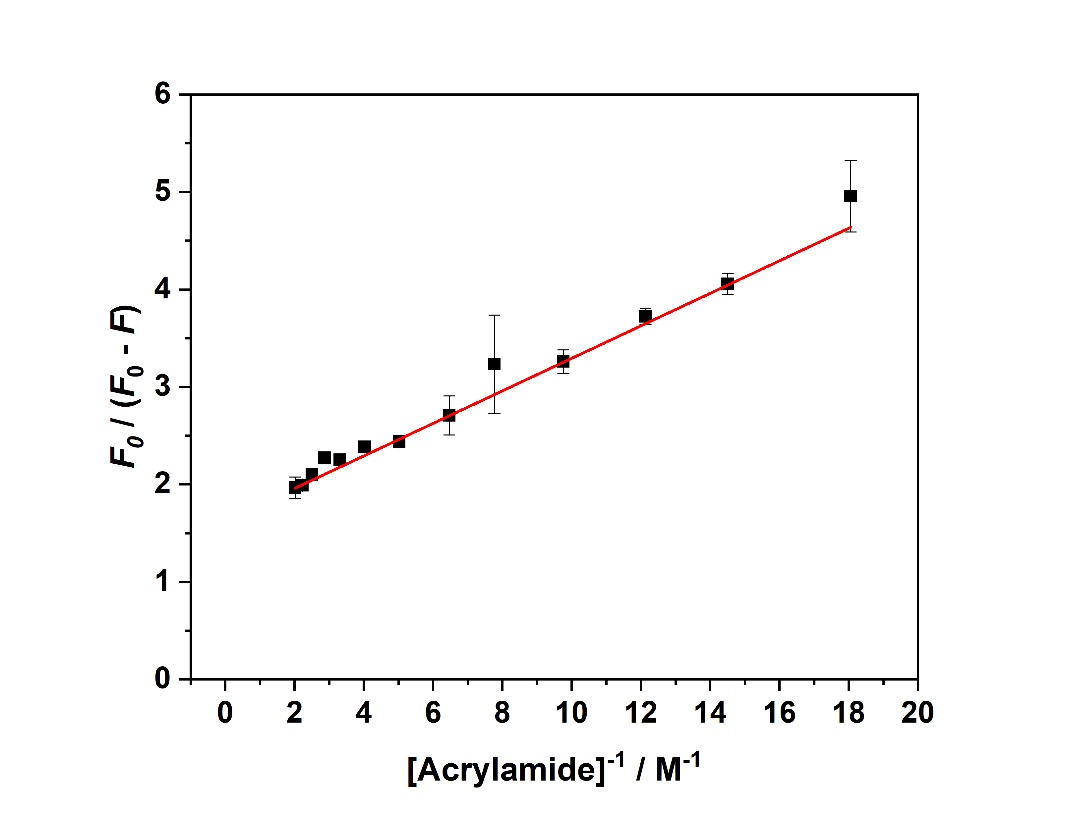


**Figure S8.** The modified Stern-Volmer plot obtained from the titration with acrylamide of a solution containing of AMP3 at 3 µM mixed with POPE/POPG vesicles at total lipid concentration of 150 µM. The experiment was performed at the temperature of 25 °C in 10 mM phosphate buffer, pH 7.4 and, using a quartz cuvette with a path length of 1 cm.

**References**

[71] A. S. Ladokhin, S. Jayasinghe, and S. H. White, ‘How to measure and analyze tryptophan fluorescence in membranes properly, and why bother?’, *Anal Biochem* **2000**, 285, 235–245, https://doi.org/10.1006/abio.2000.4773.

[72] R. Oliva, P. D. Vecchio, A. Grimaldi, E. Notomista, V. Cafaro, K. Pane, V. Schuabb, R. Winter, and L. Petraccone, ‘Membrane disintegration by the antimicrobial peptide (P)GKY20: lipid segregation and domain formation’, *Phys Chem Chem Phys* **2019**, 21, 3989–3998, https://doi.org/10.1039/C8CP06280C.

[73] R. Oliva, M. Chino, K. Pane, V. Pistorio, A. De Santis, E. Pizzo, G. D’Errico, V. Pavone, A. Lombardi, P. Del Vecchio, E. Notomista, F. Nastri, and L. Petraccone, ‘Exploring the role of unnatural amino acids in antimicrobial peptides’, *Sci Rep* **2018**, 8, 8888, https://doi.org/10.1038/s41598-018-27231-5.

[74] J. R. Lakowicz, “Principles of Fluorescence Spectroscopy,” 3rd Edition, Springer, New York, **2006.**

[75] F. Battista, R. Oliva, P. Del Vecchio, R. Winter, and L. Petraccone, ‘Insights into the Action Mechanism of the Antimicrobial Peptide Lasioglossin III’, *Int J Mol Sci* **2021**, 22, 2857, https://doi.org/10.3390/ijms22062857.
